# Impact of positive CD4 cells on event‐free survival in follicular lymphoma patients

**DOI:** 10.1002/cam4.70117

**Published:** 2024-09-09

**Authors:** Cong Li, Na Guo, Shuiyun Han, Haifeng Yu, Tao Lei, Xi Chen, Shuailing Peng, Haiyan Yang, Meijuan Wu

**Affiliations:** ^1^ Department of Lymphoma, Zhejiang Cancer Hospital Hangzhou Institute of Medicine (HIM), Chinese Academy of Sciences Hangzhou Zhejiang China; ^2^ Department of Pathology, Zhejiang Cancer Hospital Hangzhou Institute of Medicine (HIM), Chinese Academy of Sciences Hangzhou Zhejiang China

**Keywords:** CD4+, event‐free survival, follicular lymphoma, immunohistochemistry, prognosis

## Abstract

**Objective:**

Previous results about prognostic value of CD4+ T cells in follicular lymphoma (FL) remain controversial.

**Methods:**

Immunohistochemistry was used to examine expression of positive CD4 cells in 103 patients with FL 1‐3A. Early failure was described as failing to achieve event‐free survival (EFS) at 12 or 24 months.

**Results:**

There were 49 (47.6%) male and 54 (52.4%) females, with a median age of 54 years. Compared to patients with <20% of positive CD4 cells, patients with ≥20% of positive CD4 cells exhibited a significant lower risk of early failure (2‐year EFS rate: 56.7% vs 73.5%, *p* = 0.047). When patients were stratified based on positive CD4 cell combined with FLIPI, the median EFS (*p* = 0.002) and median OS (*p* = 0.007) were significantly different.

**Conclusions:**

This study demonstrated that higher expression of positive CD4 cells predicts lower risk of early failure in follicular lymphoma, and combination analysis of CD4 and FLIPI could better predict disease relapse and survival outcome.

## 1 INTRODUCTION

Follicular lymphoma (FL) is the leading type of indolent non‐Hodgkin lymphoma (NHL).^1^ The survival outcome of FL is heterogeneous, with many patients experiencing an indolent clinical course, carrying a median survival time of 10 years. However, around 20%–30% of patients can experience rapid progression or numerous relapses or transformation into an aggressive phenotype.^2^, ^3^ Previous studies revealed that disease progression within 24 months (POD24) following diagnosis was associated with poor outcomes.^4^ Several other prognostic scoring systems integrating both clinical and biologic factors were established over the years to screen for patients with high risk of disease progression or poor outcome. But these scoring systems have certain limitations. For example, the Follicular Lymphoma International Prognostic Index (FLIPI) was established in the pre‐rituximab era, thus, it exhibits limited accuracy in the current immunochemotherapy era.^5^ The FLIPI‐2 and PRIMA‐PI mainly predicts PFS, but not OS.^6^, ^7^ Lastly, the m7‐FLIPI and 23‐gene predictor used expensive and complicated sequencing and gene expression profiling, which, unfortunately, limited their clinical practice.^8^, ^9^

Emerging evidences reported that the tumor microenvironment (TME) is critical for FL onset and progression. Whole‐genome microarray of diagnostic FL biopsies uncovered that the patient genetic profile associated with desirable prognosis was enriched in T‐cell gene expression. In contrast, the genetic profile linked to worse prognosis featured macrophages.^10^ Until now, the understanding of the FL TME is still not comprehensive because of the highly heterogeneous components of TME. Among various cell subsets, T cells are the most prevalent TME components in FL, including 10%–40% of naïve and memory CD4 T cells.^11^ In the past, the tumor‐infiltrating CD4+ T cells were examined using numerous approaches of prognostic indicator analyses.^12^, ^13^ However, the conclusions were inconsistent. Hence, in this investigation, we examined the expression and location of CD4 cells in the FL tumor tissue using immunohistochemistry (IHC). We also conducted survival analysis to elucidate whether positive CD4 cell expression can predict the early failure and overall survival of FL patients.

## 2 MATERIALS AND METHODS

### 2.1 Patients

For analysis, we recruited patients with newly diagnosed FL grade 1‐3A. Patients with histologic grade 3B FL or transformation/composition of diffuse large B cell lymphoma (DLBCL) were excluded from this study. Diagnosis was based on the World Health Organization (WHO) histological guidelines, and was conducted by a highly skilled pathologist. Sufficient diagnostic formalin‐fixed, paraffin‐embedded (FFPE) lymph node tissues were obtained to examine IHC expression. Cases with negative CD4 cell expression were excluded from analysis. Treatment regimens were guided by the Groupe d'Etude des Lymphomes Folliculaires (GELF), or National Comprehensive Cancer Network (NCCN) criteria.^14^, ^15^ In terms of stage I‐II patients, initial therapy included involved‐site radiotherapy (ISRT) or ISRT+anti‐CD20 monoclonal antibody±chemotherapy, surgery resection, or observation. In terms of stage III‐IV patients, observation was recommended for patients with no indication for treatment, otherwise, systemic chemotherapy was selected. Blood cell counts, circulation biochemistry, computerized tomography scan, and bone marrow biopsy results of patients were collected. The patient response was evaluated based on conventional guidelines.^16^ All patients underwent follow‐up to monitor disease progression/relapse, retreatment, transformation, and death. Moreover, all medical events were verified using medical records. This research received ethical approval from the Zhejiang Cancer Hospital, and obtained informed consents from all subjects prior to the initiation of the study.

### 2.2 IHC staining

The paraffin‐embedded lymph node blocks prior to treatment before treatment were cut into 4‐μm sections and before application to 3‐aminopropyltrioxysilane‐coated slides. Subsequently, the specimen underwent dewaxing and blocking in a hydrogen peroxide/methanol mixture, followed by antigen retrieval via cooking the specimen in citrate in a pressure cooker. Next, the specimen underwent staining via the Vector Elite ABC kit (Ventana Medical Systems, Tucson, AZ), with subsequent diaminobenzidine chromogen staining (Biostat, Stockport, United Kingdom), as per kit directions.

### 2.3 IHC analysis

Two histopathologists independently scored all IHC results, and obtained comparable results. Tissue microarrays (TMA) were stained with CD4 according to the manufacturers' recommendations. In short, the number of CD4 positive cells per high performance fortran (HPF) (×400 magnification) was counted, and its proportion was recorded. Based on the staining degree, the CD4 positive cellular densities were categorized as follows: −, none cells/HPF; +, weakly staining cells/HPF; ++, moderately positive cells/HPF; +++, strongly positive cells/HPF. The immunoarchitectural profiles of positive CD4 cell distributions were assessed in comparison to neoplastic follicles. The “follicular” profile was manifested by peri‐ and intrafollicular CD4 positive cell predominance. Alternately, the “diffuse” profile was defined by diffusely distributed cells, with no obvious association with follicles.

### 2.4 Statistical analysis

Event‐free survival (EFS) was described as the duration from diagnosis to disease progression, relapse, therapy initiation, transformation to aggressive lymphoma, or death due to any cause. The interval between diagnosis and treatment initiation for patients with indications for treatment was less than 3 months. Early failure was described as not achieving EFS at 24 months (EFS24) in patients who received treatment immediately after diagnosis, or not achieving EFS at 12 months (EFS12) in patients under observation. Overall survival (OS) was computed from the diagnosis date till the death from any cause date or date of last contact. All time‐associated endpoints were predicted using Kaplan–Meier, and the log‐rank test was employed to compare between risk groups, as well as estimate hazard ratios (HRs) and 95% confidence intervals (*CI*). Categorical variables were analyzed using Fisher's exact or χ^2^ test, where appropriate, and continuous variables were analyzed via two‐tailed paired *t* tests. Two‐sided *p* < 0.05 was set as the significance threshold. Lastly, SPSS, version 26 was employed for all data analyses.

## 3 RESULTS

### 3.1 Patients characteristics and treatment

Between April 2004 and December 2019, 103 patients with positive CD4 cellular expression, based on IHC, were recruited for analysis. The baseline clinical profile of these patients is presented in Table 1. There were 49 (47.6%) males and 54 (52.4%) females, with a median age of 54 year (range, 27–81 years) at diagnosis. A larger proportion of patients exhibited stage III‐IV disease (66/103, 64.1%) and pathological grade 1–2 (63/103, 61.2%).

**TABLE 1:** Patient and disease characteristics.

	CD4 < 20%	CD4 ≥ 20%	Total	*p*‐value
	*N* = 42	*N* = 61	*N* = 103	
Age at diagnosis, median (range)	51 (27, 77)	58 (27, 81)	54 (27, 81)	0.896
Age ≥ 60 years, n (%)	12 (28.6%)	25 (41%)	37 (35.9%)	0.217
Male sex, *n* (%)	19 (45.2%)	30 (49.2%)	49 (47.6%)	0.841
ECOG				
0	15 (35.7%)	32 (52.5%)	47 (45.6%)	0.243
1	22 (52.4%)	24 (39.3%)	46 (44.7%)	
2	5 (11.9%)	5 (8.2%)	10 (9.7%)	
Ann Arbor stage, *n* (%)				
I–II	9 (21.4%)	28 (45.9%)	37 (35.9%)	0.013
III–IV	33 (78.6%)	33 (54.1%)	66 (64.1%)	
Histologic grade				
1–2	23 (54.8%)	40 (65.6%)	63 (61.2%)	0.307
3a	19 (45.2%)	21 (34.4%)	40 (38.8%)	
Ki‐67, *n* (%)				
≤30%	22 (54.4%)	31 (50.8%)	53 (51.5%)	1
>30%	20 (47.6%)	30 (49.2%)	50 (48.5%)	
Immunohistochemical staining, n (%)				
Bcl2+	41 (97.6%)	55 (90.2%)	96 (93.2%)	0.236
Bcl6+	42 (97.6%)	53 (86.9%)	94 (91.3%)	0.079
B symptoms, *n* (%)	6 (14.3%)	10 (16.4%)	16 (15.5%)	1
Bone marrow involvement, *n* (%)	5 (11.9%)	7 (11.5%)	12 (11.7%)	1
Extranodal involvement, *n* (%)	14 (33.3%)	24 (39.3%)	38 (36.9%)	0.678
Spleen involvement, *n* (%)	7 (16.7%)	7 (11.5%)	14 (13.6%)	0.561
Lymph node larger than 6 cm, *n* (%)	10 (23.8%)	13 (21.3%)	23 (22.3%)	0.812
Number of nodal sites ≥5, *n* (%)	19 (45.2%)	26 (42.6%)	45 (43.7%)	0.841
Elevated LDH level, *n* (%)	18 (42.9%)	16 (26.2%)	34 (33.0%)	0.091
HGB <12 g/dL, *n* (%)	11 (26.2%)	13 (21.3%)	24 (23.3%)	0.638
Elevated β2‐MG level, *n* (%)	11 (26.2%)	14 (23.0%)	25 (24.3%)	0.816
FLIPI, *n* (%)				
Low risk (0–1)	13 (31.0%)	24 (39.3%)	37 (35.9%)	0.288
Intermediate risk^2^	11 (26.2%)	20 (32.8%)	31 (30.1%)	
High risk^3^, ^4^, ^5^	18 (42.9%)	17 (27.9%)	35 (34.0%)	
FLIPI2, *n* (%)				
Low risk (0–1)	11 (26.2%)	21 (34.4%)	32 (31.1%)	0.39
Intermediate risk^2^	27 (64.3%)	31 (50.8%)	58 (56.3%)	
High risk^3^, ^4^, ^5^	4 (9.5%)	9 (14.8%)	13 (12.6%)	
PRIMA, *n* (%)				
Low risk	27 (64.3%)	42 (70.5)	70 (68.0%)	0.767
Intermediate risk	4 (9.5%)	4 (6.6%)	8 (7.8%)	
High risk	11 (26.2%)	14 (23.0%)	25 (24.2%)	
First treatment, *n* (%)				
Chemotherapy	32 (76.2%)	32 (52.5%)	64 (62.1%)	0.043
Radiotherapy	1 (2.4%)	10 (16.4%)	11 (10.7%)	
Surgery	0	1 (1.6%)	1 (1.0%)	
Observation	9 (21.4%)	18 (29.5%)	27 (26.2%)	

Abbreviations: ECOG, Eastern Cooperative oncology group; FL, follicular lymphoma; FLIPI, follicular lymphoma international prognostic index; HGB, hemoglobin; LDH, lactic dehydrogenase; β2‐MG, β2‐microglobulin.

Among 37 patients with stage I‐II, 10 patients underwent radiotherapy alone, 10 underwent chemotherapy, 9 underwent radiotherapy + rituximab‐containing immunochemotherapy, 1 underwent surgery resection, and 7 patients did not receive any form of treatment. Among the 66 patients with advanced stage, 20 patients without indications for treatment were observed. In the meantime, 46 patients with indications for treatment, received immunochemotherapy. The following induction chemotherapeutic regimens were used in routine practice: CHOP+/−R (cyclophosphamide, doxorubicin, vincristine, prednisolone, rituximab, *n* = 44), FC (cyclophosphamide, fludarabine, *n* = 1), rituximab (n = 1). 10 patients completed 2 years of rituximab maintenance therapy following chemotherapeutic initiation.

### 3.2 Predicted values of FLIPI, FLIPI‐2, PRIMA‐PI

**FIGURE 1:**
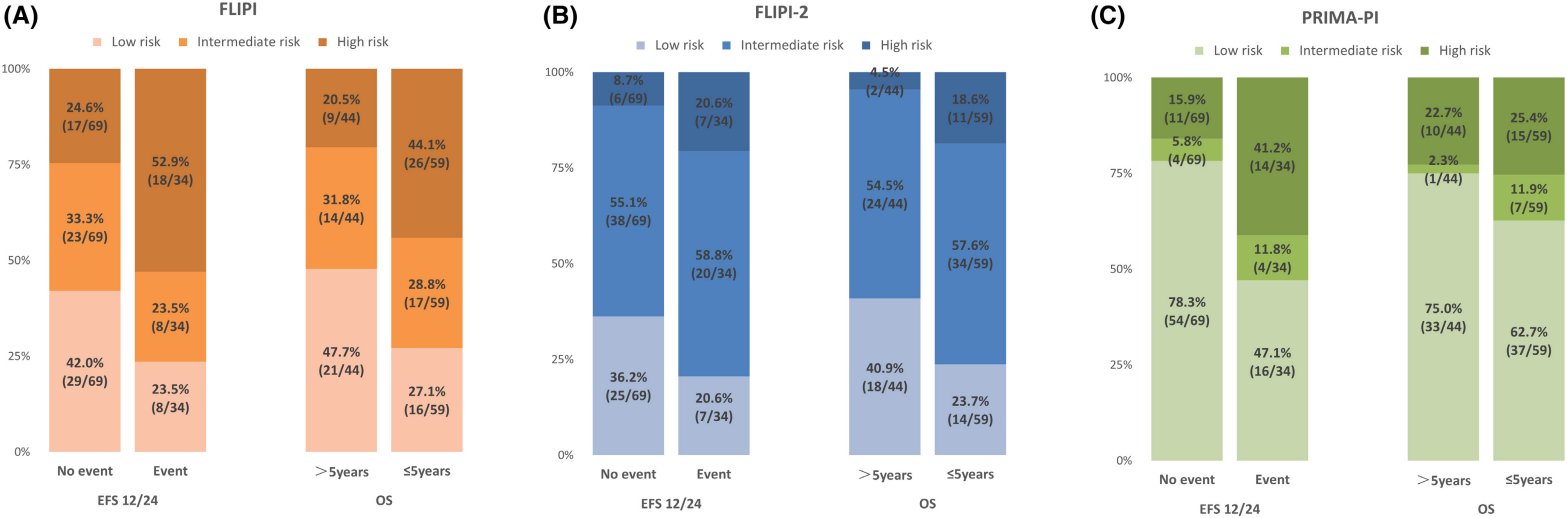
Bar graph showing the percentages of 103 follicular lymphoma patients who achieved event‐free survival (EFS) at 12 or 24 months or not and overall survival (OS) in different risk groups of FLIPI, FLIPI‐2, and PRIMA‐PI risk models.

Following a median of 52.6‐month follow‐up, 34 (33%) patients failed to achieve EFS12/24, and 44 (42.7%) patients expired. Overall, the 2‐year EFS rate was 66.6% (95% *CI*: 57.4% to 75.8%). The median OS (mOS) was not reached. The 5‐ and 10‐year OS rates were 80.1% (95% *CI*: 71.4% to 88.9%) and 71.6% (95% *CI*: 59.2% to 84.1%), respectively. We also assessed the performances of three prognostic models in the prediction of EFS12/24 and OS in our patient cohort (Figure 1). The high‐risk FLIPI identified 52.9% of patients with early failure. The high‐risk FLIPI‐2 only identified 20.6% patients with early failure, whereas, the intermediate‐risk FLIPI‐2 identified an additional 58.8% patients as early failures. Overall, similar proportion of patients with short survival outcome (<5 years) were identified by the intermediate/high‐risk of FLIPI and FLIPI‐2 (72.9% and 76.3%, respectively). By contrast, the ability of PRIMA‐PI to screen for patients with early failure was inferior to FLIPI and FLIPI‐2. In fact, the PRIMA‐PI intermediate/high‐risk only identified 52.9% patients as early failure, and 37.3% patients with poor OS (<5 years).

### 3.3 Expression of CD4

**FIGURE 2:**
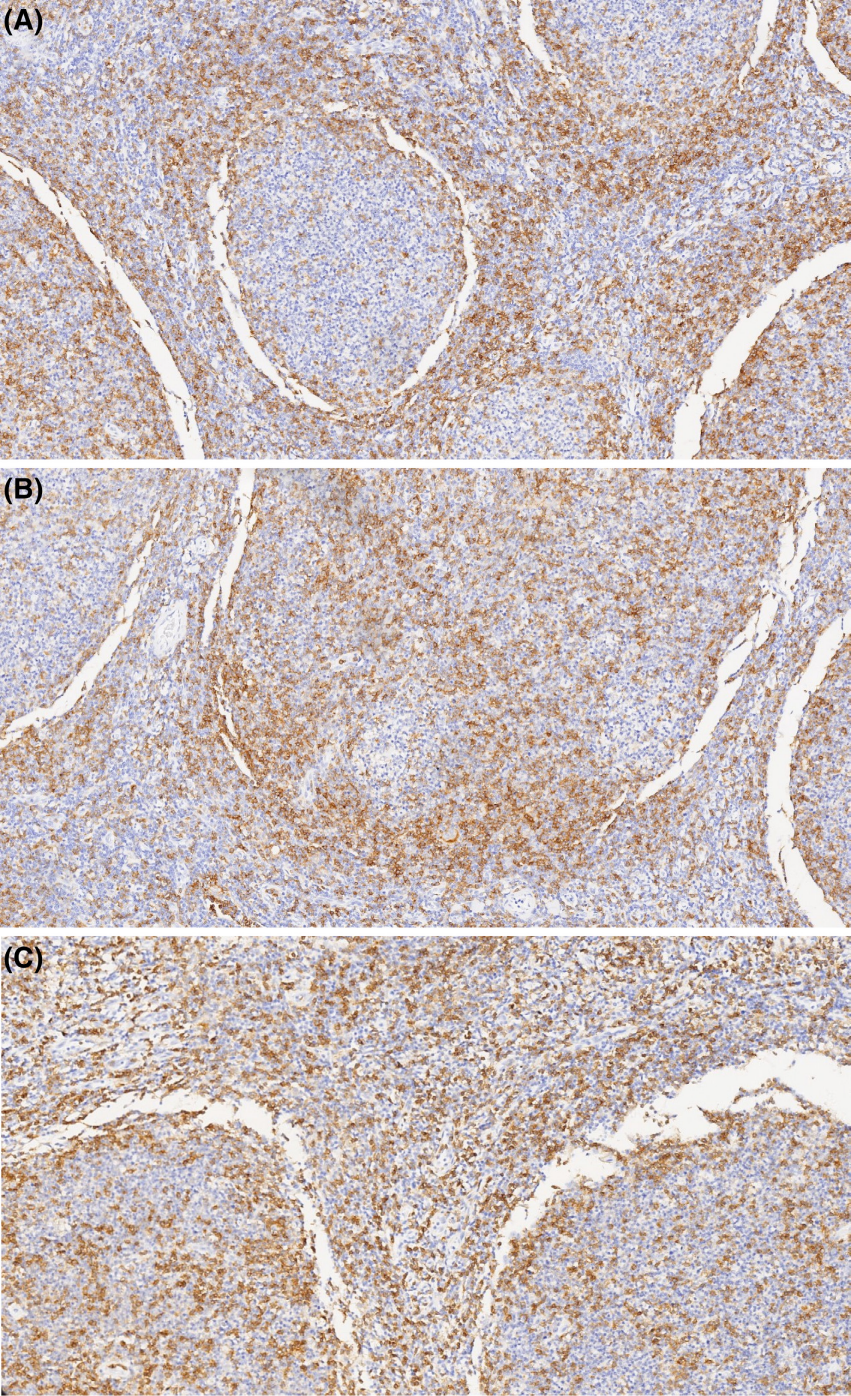
Immunohistochemical expression patterns of positive CD4 cells in follicular lymphoma. (A) Perifollicular pattern: Positive CD4 cells present mostly around the follicle. (B) Intrafollicular pattern: Positive CD4 cells present mostly within the follicle. (C) Diffuse pattern: Positive CD4 cells present diffusely with no follicle‐centered pattern defined. (IHC 100X).

The proportion of positive CD4 cells in FL tissues was distributed between 1% and 80%. The median proportion was 20%. The distribution was as follows: 1%–9%: *n* = 21 (20.4%); 10%–19%: n = 21 (20.4%); 20%–49%: *n* = 45 (43.7%); ≥50%: *n* = 16 (15.5%). The positive CD4 cell distribution was stratified into 3 main IHC profiles: perifollicular (*n* = 71, 68.9%), diffuse (*n* = 31, 30.1%), and intrafollicular patterns (n = 1, 1%, see Figure 2). As previously mentioned in the methods section, specimen exhibiting either a perifollicular or intrafollicular profile were classified as follicular profile. Thirty‐seven patients (35.9%) did not exhibit a positive CD4 intrafollicular expression. The positive intrafollicular CD4 cells were also counted in the remaining 66 patients. The distribution of positive CD4 cells was as follows: 1%–9%: *n* = 40 (60.6%); 10%–19%: *n* = 12 (18.2%); 20%–49%: *n* = 10 (15.2%); ≥50%: *n* = 4 (6%). The expression intensity of the positive CD4 cells were as follows: +, 44 (42.7%) cases; ++, 33 (32%) cases; +++, 26 (25.2%) cases. Table 2 summarizes the proportions, distribution profiles, and expression intensities of positive CD4 cells in the entire cohort and subgroups, as classified by EFS12/24 and OS.

**TABLE 2:** The expression, intensity, and distribution of positive CD4 cells in 103 follicular lymphoma patients.

	*N* (%)	EFS 12/24		*p*‐value	OS		*p*‐value
		Event (%)	No event (%)		>5 years (%)	≤5 years (%)	
Expression patterns							
Diffuse	31 (30.1%)	11 (32.4%)	20 (29.0%)	0.325	9 (20.5%)	22 (37.3%)	0.106
Perifollicular	71 (68.9%)	22 (64.7%)	49 (71.0%)		34 (77.3%)	37 (62.7%)	
Intrafollicular	1 (1.0%)	1 (2.9%)	0		1 (2.3%)	0	
Proportion of positive CD4 cells							
1%–9%	21 (20.4%)	10 (29.4%)	11 (15.9%)	0.188	11 (25%)	10 (16.9%)	0.739
10%–19%	21 (20.4%)	8 (23.5%)	13 (18.8%)		9 (20.5%)	12 (20.3%)	
20%–49%	45 (43.7%)	10 (29.4%)	35 (50.7%)		17 (38.6%)	28 (47.5%)	
≥50%	16 (15.5%)	6 (17.6%)	10 (14.5%)		7 (15.9%)	9 (15.3%)	
Expression intensity							
1	44 (42.7%)	13 (38.2)	31 (44.9%)	0.505	22 (50.0%)	22 (37.3%)	0.064
2	33 (32.0%)	10 (29.4%)	23 (33.3%)		16 (36.4%)	17 (28.8%)	
3	26 (25.2%)	11 (32.4%)	15 (21.7%)		6 (13.6%)	20 (33.9%)	

### 3.4 Relationship between positive CD4 cells and clinical features

The relationship between positive CD4 cells and primary patient clinical characteristics are listed in Table 1. No marked association was present between the positive CD4 cells and histological grade, Ki‐67 index, age, FLIPI, FLIPI‐2, and PRIMA‐PI. However, patients with low disease stage exhibited significantly more positive CD4 cells ≥20%, compared to patients with high disease stage (75.7% vs 50%, *p* = 0.011).

### 3.5 Prognostic factors for EFS and OS

Neither the CD4 expression profile nor its expression intensity displayed an impact on EFS or OS. The 2‐year EFS was: 63.5% (95% *CI*, 46.2% to 80.8%) in the diffuse profile versus zero in the intrafollicular profile versus 69% (95% *CI*, 58.2% to 79.7%) in the perifollicular profile, *p* = 0.533. The 5‐year OS rate was: 73.7% (95% *CI*, 54.7% to 92.7%) in the diffuse profile versus 100% in the intrafollicular profile versus 82.2% (95% *CI*, 72.5% to 92%) in the perifollicular profile, *p* = 0.522. The 2‐year EFS rate was: 70.3% (95% *CI*, 56.8% to 83.9%) in “+” versus 69.7% (95% *CI*, 54% to 85.4%) in “++” versus 55% (95% *CI*, 34.9% to 75.1%) in “+++”, *p* = 0.580. The 5‐year OS was: 77.8% (95% *CI*, 64.7% to 90.8%) in “+” versus 83.4% (95% *CI*, 69.9% to 96.9%) in “++” versus 76.2% (95% CI, 50.9% to 100%) in “+++”, *p* = 0.696.

**FIGURE 3:**
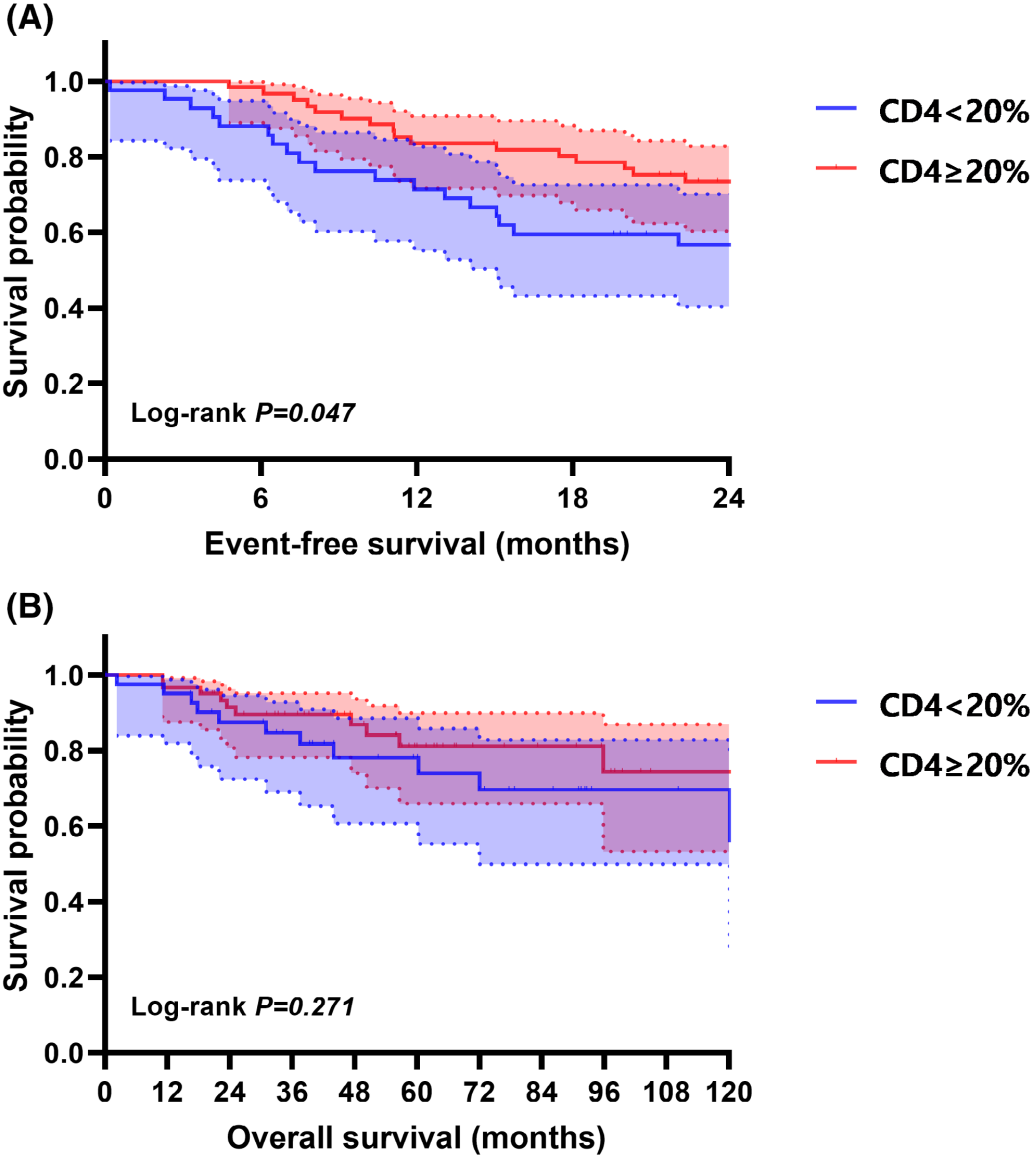
Event‐free survival (EFS) and overall survival (OS) in patients with FL in relation to positive CD4 cells (<20% vs ≥20%).

Compared to patients with <20% of positive CD4 cells, patients with ≥20% of positive CD4 cells exhibited a significantly lower risk of early failure (2‐year EFS rate: 56.7% (95% *CI*, 41.5% to 71.8%) versus 73.5% (95% *CI*, 62.3% to 84.6%), *p* = 0.047). OS, however, did not differ significantly between the two patient cohorts (5‐year OS: 78.2% (95% *CI*: 64.5% to 91.8%) versus 81.1% (95% *CI*: 69.4% to 92.7%), *p* = 0.271) (Figure 3). Alternately, compared to the patients at low/intermediate risk of FLIPI, patients at the high risk FLIPI exhibited a significantly higher risk of early failure (2‐year EFS rate: 76.4% (95% *CI*, 66.3% to 86.5%) versus 47.3% (95% *CI*, 30.4% to 64.2%), *p* = 0.001) and worse OS (5‐year OS: 83.8% (95% *CI*: 73.9% to 93.7%) versus 74.3% (95% *CI*: 58.7% to 89.9%), *p* = 0.031).

**FIGURE 4:**
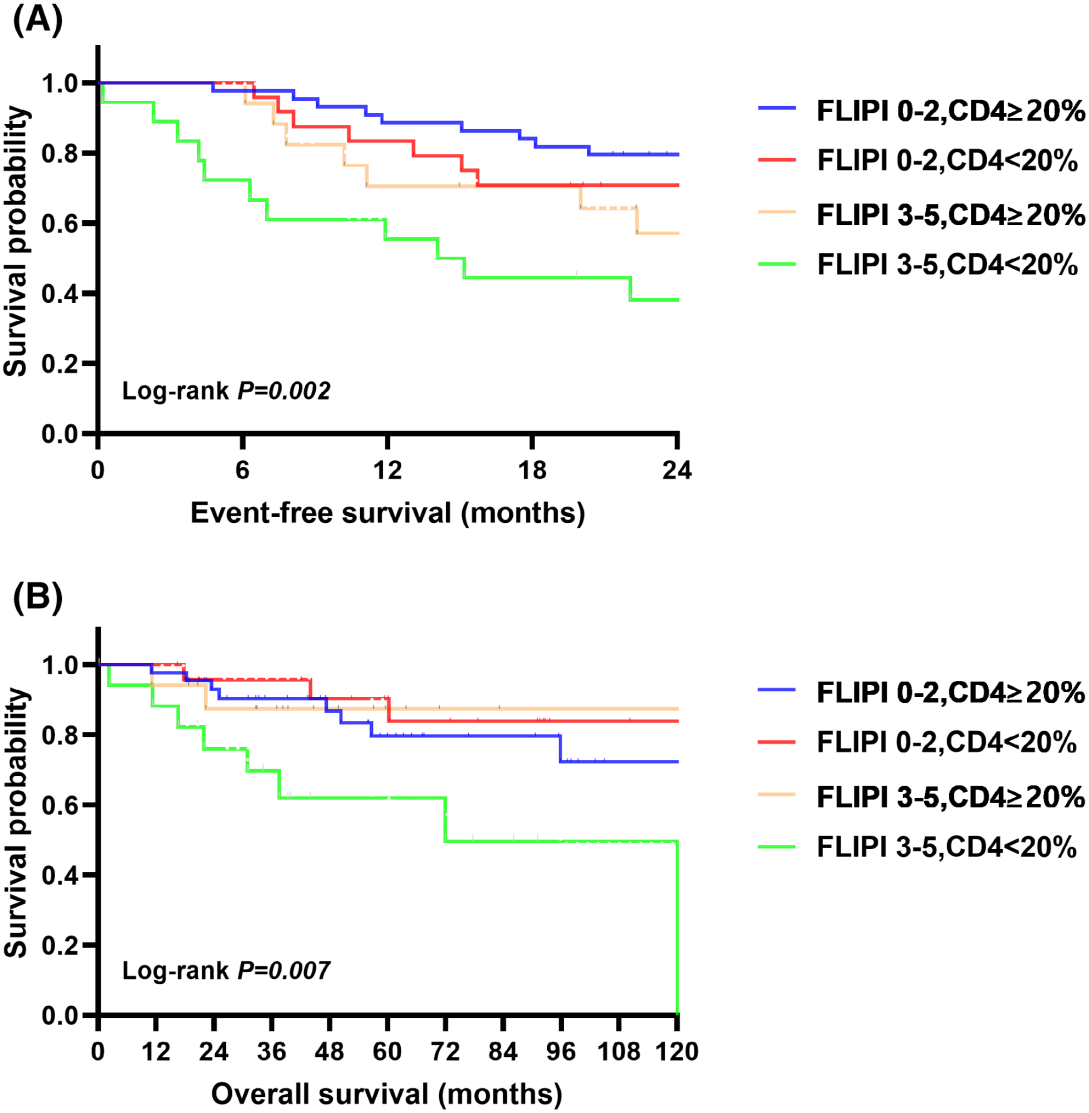
Event‐free survival (EFS) and overall survival (OS) in patients with FL based on positive CD4 cells combined with FLIPI risk model.

Given the prognostic value of positive CD4 cells and FLIPI, we further classified all patients into 4 groups, based on these two factors: group 1 (FLIPI score 0–2 + positive proportion of CD4 cells ≥20%, *n* = 44), group 2 (FLIPI score 0–2 + positive proportion of CD4 cells <20%, *n* = 24), group 3 (FLIPI score 3–5 + positive proportion of CD4 cells ≥20%, *n* = 17), group 4 (FLIPI score 3–5 + positive proportion of CD4 cells <20%, *n* = 18). The median EFS (mEFS) (*p* = 0.002) and median OS (*p* = 0.007) were differed significantly among these four groups, and patients in group 4 exhibited the largest risk of early failure (mEFS 14.1 months, 95% *CI*: 7.3 to 20.9 months) and poorest OS (mOS 72.0 months, 95% *CI*: 33.0 to 111.0 months) (Figure 4).

## 4 DISCUSSION

Besides common clinical parameters, lymphomagenesis of FL are highly dependent on interactions with a specific and complex TME.^17^ The TME of FL is composed of follicular dendritic cells (FDCs), follicular regulatory T (Treg) cells, tumor‐infiltrating T cells (TILs), lymphoma‐associated macrophages (LAMs), and immune checkpoint‐related immune cells.^11^, ^18^ Among these components in FL TME, CD4+ TILs play an important role. They can impair the recruitment and activity of essential transduction proteins to the immunologic synapse.^19^ They also exhibit drastically dysfunctional motility, relative to healthy TILs from the reactive tonsils.^20^ Cytotoxic CD4 T cells can eliminate senescent fibroblasts, presenting their broad immune surveillance capability.^21^ Low CD4 expression or absence of intrafollicular memory CD4+ T cells can be helpful to predict patient risk for POD24.^22^, ^23^ Therefore, we selected CD4 as a potential prognostic indicator in this study.

Most previous studies examining the relationship between CD4 expression and patient survival are controversial.^24^ Using IHC assessment, Abigail M Lee al. demonstrated that CD4+ stained cells were significantly more common in FL patients who survived for over 15 years, compared to their short‐lived counterparts.^25^ In the Wahlin et al. study, a tissue microarray was generated from 70 FL biopsies, and cellular subsets were quantified via computerized image analysis. The results revealed that a large proportion of intrafollicular CD4+ cells, as well as elevated CD4 follicular/interfollicular ratios, are strongly correlated with worse prognosis in FL patients.^26^ Similarly, the Anna Maria study also employed tissue microarrays, multiplex immunofluorescence, and Shannon's entropy to test FL tissues in 127 patients, who received different treatments. Based on her results, the enhanced immune infiltrate population diversity, including CD4, is strongly associated with improved OS.^27^ Yang et al evaluated 82 FL patient tumor tissues using Mass cytometry (CyTOF). When they analyzed patients with FL grade 1–2, they reported that increased T cells (including CD4+) significantly correlated with a favorable survival. But there is no similar prognostic significance in patients with FL grade 3.^28^ Sayako Yuda et al. examined variables that influenced the treatment‐free period in FL patients using a watch‐and‐wait approach. They reported that elevated interfollicular CD4+ quantities in the TME were a significant adverse factor in univariate analyses.^29^ However, other studies observed no prognostic value for CD4 at both the protein and mRNA levels.^30^, ^31^ We speculate that patient selection and different testing or analytical methods may have affected the final results in the aforementioned publications. To better illustrate the impact of CD4 expression on patient survival, we merely focused on cases with a positive CD4 expression, and excluded cases without CD4 expression. Additionally, we utilized IHC to evaluate the CD4 expression within FL tumor tissues as IHC is the most widely employed technique in both clinical and pathologic practices, and it has the characteristics of convenience, simplicity, and repeatability.

Another possible reason for the heterogeneity within the aforementioned results is the definition of the study end point. The treatment regimens for FL 1‐3A were heterogeneous. A ‘watch and wait’ approach is a potential strategy for asymptomatic FL patients.^32^ However, some patients may experience disease progression or transformation, and may require immediate anti‐tumor intervention. Similarly, some symptomatic FL patients can experience disease relapse after immunochemotherapy. The interval between the start of immunochemotherapy and disease relapse or the interval between disease diagnosis and first‐line treatment onset is an essential indicator of early failure. Therefore, it is unreasonable to employ the same study endpoint to define the early failure events for all patients, including both early and advanced FL stages, as was performed in the aforementioned studies.^26^, ^31^ In order to avoid this unreasonable issue, we defined early failure as the failure to achieve EFS12 for observed patient or failure to achieve EFS24 for treated patients.

Our results revealed that an elevated positive CD4 expression indicated a lower risk of early failure in FL patients. In terms of using FLIPI‐2, PRIMA‐PI, and FLIPI to predict patient EFS or OS, high‐risk FLIPI was far superior in identifying patients with poor survival (44.1%) and high‐risk of early failure (52.9%), even though the FLIPI scoring system was developed in the pre‐rituximab era. When patients were classified by FLIPI and the positive proportion of CD4 cells, patients with FLIPI score 3–5 and positive CD4 cells <20% were significantly associated with early failure and inferior OS.

Prior studies revealed that elevated CD4+ infiltration is more prominent in patients without B symptoms, low performance status, and enhanced transformation risk.^26^, ^33^ In addition to CD4 quantity, the location of positive CD4 cells is another essential factor in host response to FL.^34^ The Toma et al study demonstrated that 98.4% of CD4 are present outside the neoplastic follicles.^33^ The spatial distribution of CD4‐positive T‐helper cells is known to be highly discriminatory in predicting early FL transformation.^35^ In this study, the positive CD4 cell expression profile primarily included perifollicular and diffuse profiles. Moreover, positive CD4 cells ≥20% occurred more commonly in patients with low disease stages than with high disease stages. However, the positive CD4 cell expression profile and intensity showed no impact on EFS12/24 or OS.

However, a critical question arises while analyzing these results. FL pathogenesis and prognosis are affected by various immune cell compositions working simultaneously in the TME, rather than being dictated by the action of an individual immune cell subset.^36^ Therefore, it is difficult to elucidate the entire function and role of the TME by examining a single cell or cytokine. Some modern technologies, including single‐cell RNA sequencing, can identify previously unknown cell subsets and define their distribution patterns and expression degree, however, the clinical and biological significance of TME is not entirely clear.^13^, ^37^, ^38^ The important significance of our study is that by a simple and affordable testing method for a regular histopathological index, we can better screen patients with early failure or poor outcome at the time of diagnosis.

## 5 CONCLUSION

This study demonstrated that elevated CD4 positive cell expression in FL patients has a lower risk of early failure. Hence, a combination of positive CD4 cells <20% with high risk FLIPI can better predict patients with early failure and poor OS.

## AUTHOR CONTRIBUTIONS

**Cong Li:** Conceptualization (equal); data curation (equal); visualization (equal); writing – original draft (equal). **Na Guo:** Formal analysis (equal); investigation (equal); resources (equal); writing – original draft (equal). **Shuiyun Han:** Data curation (equal); investigation (equal); project administration (equal); resources (equal). **Haifeng Yu:** Data curation (equal); investigation (equal); validation (equal). **Tao Lei:** Formal analysis (equal); resources (equal); supervision (equal); validation (equal). **Xi Chen:** Investigation (equal); resources (equal); validation (equal). **Shuailing Peng:** Formal analysis (equal); software (equal); validation (equal). **Haiyan Yang:** Conceptualization (equal); project administration (equal); supervision (equal); writing – review and editing (equal). **Meijuan Wu:** Conceptualization (equal); methodology (equal); project administration (equal); supervision (equal); writing – review and editing (equal).

## FUNDING INFORMATION

This work was supported by the medical and health research project of Zhejiang province (2021KY105).

## Data Availability

All data generated during this study are included in this published article. The datasets used during the current study are available from the corresponding author on reasonable request.
